# Benefits and challenges experienced by participants on long-term methadone maintenance treatment in China: a qualitative study

**DOI:** 10.1186/s12916-023-03203-z

**Published:** 2024-01-08

**Authors:** Xijia Tang, Wenxue Xiong, Wen Chen, Chijie Wang, Hexuan Wang, Boyu Li, Zirong Zhang, Li Ling

**Affiliations:** 1 Department of Medical Statistics, School of Public Health, Sun Yat-Sen University, No.74 Zhongshan 2Nd Road, Yuexiu District, Guangzhou, Guangdong PR China 510080; 2 School of Mathematics, Sun Yat-Sen University, Guangzhou, Guangdong PR China; 3 Clinical Research Design Division, Clinical Research Center, Sun-Yat Sen Memorial Hospital, Sun Yat-Sen University, Guangzhou, Guangdong PR China 510120

**Keywords:** Methadone maintenance treatment, Long-term retention, Benefits, Challenges, Qualitative study, China

## Abstract

**Background:**

Methadone maintenance treatment (MMT) has been implemented in China for nearly two decades, with a significant decrease in the number of participants in recent years. However, there is a lack of comprehensive research focusing on the long-term effectiveness in the context of this decline, especially from the perspectives of MMT participants themselves. This study aims to address this gap by examining the benefits and challenges experienced by long-term MMT participants in China, to uncover potential causes of the decrease in participant numbers and to improve the effectiveness of the program.

**Methods:**

We conducted semi-structured interviews with 21 long-term MMT participants (treatment duration ≥ 5 years) recruited through purposive sampling from 6 MMT clinics in the Guangdong Province, China, between December 2021 and August 2022. Thematic analysis was employed to analyze the transcribed interviews. Two analysts independently coded the data, and a third researcher double-coded 20% of transcripts to ensure intercoder reliability.

**Results:**

Overall, participants corroborated the notable decline in MMT participants during their long-term MMT, citing death, arrest, and self-perceived abstinence from heroin, as their perceived driving factors. They reported positive changes in their health, family relationships, and social functioning. However, they identified economic hardship as their greatest challenge associated with MMT, further exacerbated by other barriers including the conflict of clinic opening hours and working schedules, discrimination from employers, and COVID-19-related restrictions. Additionally, participants identified issues with dose adjustment and emergency treatment continuation.

**Conclusions:**

This study outlines the overall improvement in the quality of life of long-term MMT participants. However, it highlights the need for official guidelines for dose adjustment and emergency treatment continuation as well as the provision of health education, job referrals, and flexibility of clinic opening times to facilitate the return to society receiving participants. Establishing a follow-up mechanism for those receiving MMT is also recommended to prevent relapses to heroin and other illicit substances.

**Supplementary Information:**

The online version contains supplementary material available at 10.1186/s12916-023-03203-z.

## Background

Illicit drug use is a global public health concern that affects millions of people worldwide [1]. In China, heroin was the dominant illicit drug in the late 1990s, resulting in the launch of a series of harm-reduction interventions, including methadone maintenance treatment (MMT) [2]. The program was rapidly scaled up nationwide over the past two decades, ranked the world’s largest MMT program, covering 30 of 31 provinces and serving approximately 300,000 users [3].

Throughout its 20-year implementation, MMT has shown to be effective in reducing drug use, HIV infection, and criminal behaviors, while improving family relationships of participants [4]. Specifically, the rate of new HIV infections of Chinese MMT participants declined from 0.95% in 2006 to 0.07% in 2021, and the rate of positive urine morphine tests dropped from 27.2% in 2008 to 9.6% in 2021 [5]. These achievements are important for enhancing the health and social well-being of people who use drugs (PWUD) and reducing the negative impact on society.

Heroin has become less dominant as an illicit drug both globally and in China in recent years due to concerted efforts [6]. According to the Chinese National Narcotics Control Commission [7, 8], people who use heroin (PWUH) accounted for only 37% of all registered all registered PWUD in 2021, down from 81% in 2004. As a result, the pool of potential MMT participants has shrunk since there are no other endemic opioids. The Chinese Center for Disease Prevention and Control (China CDC) has reported a continuous decrease in MMT participants since 2011, with a 56.2% decline by 2020, from 208 to 91 thousand [9]. However, additional barriers such as low retention and high drop-out rate of participants may have also contributed to the decrease [10]. These trends not only jeopardize the quality and sustainability of the MMT program but also risk misusing valuable resources and demotivating staff members.

Previous studies have primarily focused on the effectiveness of MMT by examining specific indicators, such as the HIV/HCV prevalence of participants with relatively short treatment duration [11, 12]. However, to gain a comprehensive understanding of the MMT program’s effectiveness over the past two decades, it is crucial to consider the perspectives of participants who have received long-term treatment. Their experiences can provide representative and reliable insights, which can, in turn, help to address the existing challenges.

Therefore, this study aims to explore the benefits and challenges experienced by participants after long-term MMT in the context of the constant decline in MMT population. The findings of this study could provide references for interventions and policy-making to ensure the effectiveness of MMT.

## Methods

### Study design

This is a qualitative study using semi-structured interviews with participants who have been receiving long-term MMT (over 5 years) to obtain rich information on MMT in China from the perspectives of participants.

### Study setting and participant recruitment

The interviews were conducted in six clinics from five cities in Guangdong Province, China, from December 2021 to September 2022. This setting was selected because it previously homed the most people who use drugs (PWUD) in China, accounting for over 30% of the total number, and is now running the largest number of MMT clinics (75) [9, 13]. The purposive sampling strategy was applied to explore in-depth information from participants with various experiences [14]. Participants were eligible if they were (1) over 20 years old, (2) enrolled in MMT for more than 5 years, and (3) able to talk with others for 20–40 min.

### Data collection

The original topic guide was developed based on the aim of this study. We conducted 4 pilot interviews in December 2021 and adjusted the topic guide accordingly (see Additional file 1). Interviews were conducted face-to-face in a private room in the MMT clinic with consent given by one researcher (XT). The consent form was provided to participants prior to the interview, informing them that their confidentiality would be ensured and they were free to withdraw at any time in the process. The duration of interviews varied from 17 to 64 min, with an average of 32 min. All the interviews were audio recorded and transcribed verbatim in Chinese. The transcribed texts were then translated into English by another researcher (HW). Interviews were continued until the information saturation was reached, meaning no additional information could be received from extra participants [15]. No identifiable information, such as names, telephone numbers, or home addresses, was collected for analysis. Participants who completed the interview received the incentive of 50 Yuan (around 7 US dollars) in cash.

### Data analysis

The qualitative analysis was conducted using a phenomenological approach, specifically interpretative phenomenological analysis (IPA). This approach aims to capture the essence of a participant’s subjective experience in their own terms, delineating key elements [16]. We applied IPA in this study to focus on the meaning of the experiences of long-term MMT participants and explore its significance for this group. Two members of the research team (XT, WX) independently reviewed transcripts and coded the segments relevant to the research question. A third member (HW) double-coded 20% of transcripts to ensure intercoder reliability. The initial codes were then sorted into emerging themes, which were then reviewed, and a new theme would be created if some code did not fit the existing ones. Finally, the final form of each theme was mapped and illustrated.

The analysis was conducted using the NVivo 12.0 software.

### Ethics statement and consent

This study was reviewed and approved by the Institutional Review Board of the School of Public Health, Sun Yat-sen University, Guangzhou, China (No. 2020–39). Before the interviews commenced, the aim of the interviews was verbally explained to participants, and written and oral informed consent was obtained from each participant.

## Results

A total of 21 MMT participants from 6 clinics were interviewed, 14 of whom (67.7%) were males. Their average age was approximately 48 ± 6.1 years old, and they had a drug user history of 13.9 ± 4.9 years. The duration of MMT ranged from 5 to 16 years, with a mean value of 12.0 ± 3.7 years. The daily dose they took had a median of 47 mg. The detailed characteristics of these variables are listed in Table 1.

**Table 1:** The characteristics of interviewed MMT participants

**Variable**	**Measure**	
	**No. (%)**	
Sex	14 (67.7)	
Male	7 (33.3)	
Female		
	**Mean ± SD**	**Median (Q1, Q3)**
Age (years old)	48.1 ± 6.1	46.0 (44.0, 51.0)
Duration of drug use (years)	13.9 ± 4.9	14.0 (11.0, 18.0)
Duration of receiving MMT (years)	12.0 ± 3.7	14.0 (10.0, 15.0)
	**Mean ± SD**	**Median (Q1, Q3)**
Daily methadone dose (mg)	65.6 ± 52.1	47.0 (20.0, 125.0)

### Changes in the number of MMT participants

With the decline of PWUH and MMT participants in recent years, we asked the participants about their views on this changing trend. Most of them acknowledged the decline and proposed several possible factors, including death, arrest, and self-perceived abstinence from both heroin and methadone. There may also be people using other illicit drugs as an alternative after quitting MMT.

Many of them died, some of which were still on MMT at that time. Usually, they died of cancer, mostly progressed from hepatitis C, because they had no money to get treated. The majority of us had HCV infection, but few would seek medical help due to the financial hardship.

[P4-male-50 years old] (Note: P4 means the 4th participants interviewed）

There are fewer and fewer people coming here for MMT. I think half died, and half got clean from MMT. Many friends of mine died. Only this year, I have heard that several friends passed away because of illness. They suffer from various kinds of diseases, such as heart attacks, hypertension, and liver or lung cancer due to their long history of drug use. Some of them were very young, just around 40–50 years old.

A lot of people around me have quit since they were totally clean. They have probably been treated for over ten years, since when the clinic opened.

Those who should have come to the MMT clinic had already come. Since heroin is no longer popular, there are no new participants enrolled. I did not know anyone who was still on heroin but had not attended MMT.

[P9-male-46 years old]

I saw fewer and fewer people in the clinic. Some of them get clean, while a few of them may have relapsed. over half of them died of diseases. They injected drugs into veins previously, which were so harmful, and they did not do body examinations either. Some people choose to use other drugs, such as methamphetamines. Being arrested is another reason. I think it’s good for them because compulsory rehabilitation can help them detox.

[P18-male-48 years old]

### Improvements in personal life

At the individual level, participants reported positive changes in their personal lives, including better health status, closer family relationships, and improved social functioning.

#### Physical and mental health status

The improvement of health is the most noted change by participants. Because of their long history of drug use, their health was poor before attending MMT, especially for those who were living with HIV. Symptoms including high fever, chills, swelling, and insomnia were common when they used heroin. MMT helped them relieve these symptoms and improve sleep quality and appetite.

After attending MMT, my health is much better. Before that, I had to inject drugs every day, and you can see swelling and bruising all over my body. It just looked so scary.

[P4-male-53 years old]

I felt more energetic after being treated. My sleep and appetite are much improved. It surprised me that methadone works so well.

[P1-female-42 years old].

As for their mental health status, being irritable, anxious, and frightened were experienced by many of the participants previously. This hindered both their health and social function. Years of MMT made them able to work and function like non-PWUDs, which further benefited their mental health.

At least, I am more mentally relaxed (after MMT). I don’t act as irritated as before and can think about things more deeply and calmly. Before attending MMT, I would never have been this peaceful, as my whole mind was about drugs, rather than working. I’ve been in the catering industry for 20 years, and I’ve been on heroin for more than half of 20 years. I had to frequently ask for leave during working time due to the strong craving, otherwise, I couldn’t bear it. I felt shameful myself and people would not hire me anymore either.

[P10-female-48 years old]

I feel much better. My assessment of MMT is that I was truly reborn after 2006 (when I attended MMT), my new life started then. Before that, I was muddling through. Only when I received MMT and got off the heroin, did I find my true character.

[P7-male-42 years old]

#### Family relationships

Improved family relationships were another dominant change reported by participants. Being addicted to heroin had a great negative influence on the interpersonal relationships of the users, especially with family members. They had to depend on their families for survival as they had spent all their savings on drugs and struggled with work due to the cravings, which drove some families into poverty. Many of them were, therefore, separated from their families, resulting in challenging living situations. In contrast, participating in MMT not only curbed their cravings, which saved money that might have been spent on drugs but also improved their working capacity. Both of these reduced the heavy financial reliance on the family.

The relationship with my family has had such a great improvement (after MMT). Before that, family members just isolated me, because so many years of drug use made them completely lose confidence in me. However, they found me not taking heroin anymore after attending MMT, and they started to accept me again. I am now living with my family.

[P6-male-42 years old]

I always asked the family for 100 Yuan a day when I was on heroin, so there must be quarrels and arguments. Now everything is different. They are willing to give me 10 Yuan per day for treatment as it is affordable and they know I am doing the right thing. They once blamed me because I did something wrong. Now I do not ask them for so much money, and I do not have any income, they will support me anyway.

[P9-male-46 years old]

#### Social functioning

According to the participants, MMT also played a role in the recovery of their social functioning. They were able to access employment or enjoy life after treatment, and some of them became volunteers for narcotics and HIV control. This in turn improved their mental health, family relationships, and reduced the financial burden, forming a virtuous circle for a better quality of life.

I have a job now, which released my financial burden, so it was quite worth coming to MMT. I’m running my small business, selling handbags, clothes, and so on. I can earn one to two hundred Yuan a day, and that’s enough for a living. I also work as a community volunteer, which is not profitable, but I feel so happy and fulfilled. Living a normal life is so comfortable.

[P5-female-52 years old]

Having methadone here for treatment is legal, at least the police won’t come to arrest me. Therefore, I have no worries, I am free and troubleless. I can walk on the street with my head up.

[P15-male-50 years old]

### Challenges encountered during long-term treatment

During their long treatment duration, the participants also experienced challenges and difficulties. They concluded the challenges they were facing mainly from aspects of treatment and their personal lives.

### Treatment-related challenges

#### Restriction on daily life

Being time-restricted was the most mentioned challenge. Participants have to go to the clinic every single day, and its opening times usually overlap with working hours. This made participants unable to attend work on time and created problems for those who were job-seeking. This challenge may be exacerbated with longer treatment.

We need to take this medicine every day, so we cannot leave this city. I need to take care of my parent at present, but after they pass away, I still want to travel around. However, it is impossible, and the referral is also very troublesome. I seldom go to the countryside, where my hometown is. I just can’t arrange the treatment and travel at the same time.

[P5-female-52 years old]

It’s hard to find a job. I need to come to the clinic at 8 am which makes it impossible to start work at 8 am as required, I can’t make it. It would be better to have a job that has the evening shift, but I have not found one now. The working time must adapt to the opening time of the clinic, as you can’t be late every day, and the boss won’t recruit you.

[P18-male-44 years old]

Long travel distances were also challenging for MMT participants, especially those living in remote areas. This increased the time-spent and cost of accessing the treatment and resulted in negative impacts on work and finances. However, most of them had no choice as there were few clinics in the whole district or city.

When I worked in city A, I needed to go to a clinic in city B for treatment, as that was the nearest one. It took me more than one hour by bus and I needed to transfer twice. I finish the evening shift at 8:30 a.m. and can be back home at 11 p.m. I try to look for jobs that are closer to the clinic, that’s the only thing we can do. This problem has a huge impact. Also, when there is typhoon, rain, and so on, it could be more bothering.

[P18-male-44 years old]

#### The uncertainty of the treatment continuity

Ensuring treatment continuation for participants during emergencies, such as hospitalization, remained uncertain, and was brought to the foreground during the COVID-19 pandemic. Although most participants did not report issues with accessing MMT when quarantined during the pandemic, as the treatment could be obtained via door-to-door delivery, the uncertainty still triggered their anxiety and depression. Several participants who experienced hospitalization complained about complications with the methadone delivery process.

I needed to go to the clinic in another district since this area was in lockdown. I used to come here twice a day, and I had to drink all the methadone at one clinic because it was too far away. My main anxiety was that I still needed to pay for rent when my store closed, and how I could ensure the treatment was being quarantined. I called the doctor before the lockdown and was told that I could go to another clinic, which made me feel much better.

[P12-male-49 years old]

The biggest problem is that I can’t get methadone when I am in the hospital. Previously, I can ask the doctor for proof, which allowed my family to get methadone for me. But this is not allowed anymore. The last time I was hospitalized, I still needed to come to the MMT clinic every day, which took me an hour by taxi. This problem must be solved. I don’t even know what will happen if I get sick again in the future. Many people like me, will feel like dying if they can take methadone on time. It’s extremely torture as we’re already in poor health status. It’s essential to have someone to deliver the methadone to us, anyone will do, as long as we can get it. Because we have been treated for so long, we can’t bear the consequence of pausing it for days.

[P7-male-59 years old]

#### The lack of official guidelines for dose adjustment

Nearly all participants have adjusted their daily doses multiple times during long-term treatment. Specifically, they would increase their dose when they were ill and those who are living with HIV, cancer, or suffer from pain need a higher dose. Nevertheless, most participants had a strong desire to decrease their dose and were managing to do it. Inappropriate speed or frequency of dose tapering led to side effects, mostly insomnia. However, there are no official guidelines for dose tapering in China at present.

I drink about 130 ml currently. It didn’t used to be that much, but I’m slowly adding it because I can’t get sleep and I am taking the antiviral drugs for HIV treatment. These medications are hedged with methadone, that’s why I drink much. Each time I added more I would sleep a little better, otherwise, I couldn’t sleep and would feel depressed. Once I feel better, I will still taper the dose. People I know are also trying to do so. Some of them decreased it to 2 or 3 ml and then quit MMT. They have never come back to the clinic. I want to quit MMT like them as well.

[P12-male- 49 years old]

### Personal challenges

#### Economic hardship

Economic hardship was the dominant challenge for this community, according to the participants. Some were unable to work due to illness or old age and had to completely depend on family or basic living allowances. Although MMT is relatively inexpensive at 10 Yuan per day, some participants still felt it was unaffordable. In addition, those who were employed experienced difficult choices because of time conflicts between the opening hours of clinics and commuting time, resulting in employment discrimination. These all added extra pressure to their economic hardship.

Many of these participants, spent everything out on drugs and are also too old to work. Their family isolated them as well. It is really hard for them to survive. They needed to borrow 10 YUAN a day for MMT, and when they got 10 YUAN, the clinic was already closed. So, they have to help smugglers to sell drugs to get money and heroin, which is not good for society and themselves. But they had no choice.

[P5-female-52 years old]

#### Social discrimination

Experiences of discrimination were not unusual for MMT participants, especially when hunting for jobs, as they would not get employed when their history of drug use was known. Also, socializing with others could be challenging, as others might avoid interacting with them due to their identification as drug users.

If the employers knew I’d used the drug before, they wouldn’t hire me. Even if I’m not on drugs now, they won’t want me anyway. The public all look down on us and discriminate against us.

[P20-male-46 years old]

In light of these challenges, participants provided several suggestions for both MMT clinics and public sectors of the government. All suggestions mentioned above are summarized in Additional file 1: Table S1.

## Discussion

This qualitative study explored the benefits and challenges experienced by long-term MMT participants in China in the context of the declining MMT population. Participants in this study corroborated the declining trend and considered death, arrest, and self-perceived abstinence as the main causes. In terms of individual treatment, they reported notable improvements in health, family relationships, and social functioning. Despite the positive changes, they also encountered several challenges such as the heavy financial hardship and conflicts between working time and the clinic opening times.

Over the past decade, the number of MMT participants has significantly decreased. According to China CDC [9], there was an almost 20% decrease in MMT participants in 2020, with Guangdong experiencing a sharper decrease compared to other provinces, possibly due to its larger MMT population base and efforts made to heroin control. The participants of this study corroborated the decline and considered death caused by illness as the leading reason. A long history of drug use is strongly associated with life-threatening diseases, such as HIV and cancer, which can hugely impact health among this group. For instance, over 60% of Chinese MMT participants were infected with HCV [11], which could result in liver cancer, the most-mentioned death cause in line with our participants. The risk of getting severe illness will further increase as they get older. Another reason for this decline was the voluntary withdrawal from MMT. Some participants cited no longer being dependent on heroin and chose to quit MMT permanently. However, research suggests that these individuals may have an increased risk of relapse to alternative drugs such as methamphetamine, due to inadequate perception of the benefits of MMT [17, 18]. This situation has also been observed in other countries, including Vietnam and Canada, and has raised great public health concerns [19, 20]. This alerts us to pay more attention to those who voluntarily quit MMT, as well as to strengthen education to existing participants, to prevent relapse, especially to synthetic or new drugs among these groups.

In contrast to the participant’s views, being arrested and sent to the compulsory rehabilitation center was ranked as the top cause of the decrease in Chinese MMT participants during the decade by China CDC [9]. This was only reported by a small proportion of long-term participants in our study, which may be because participants who have undergone long-term MMT are more likely to experience improvements in their social functioning, which resulted in less likely to engage in criminal behavior, compared to the short-term participants.

Long-term participants experienced a range of benefits during their treatment. At the individual level, positive changes in both physical and mental health were most vocalized, which aligns with other studies [21, 22]. Most participants were in poor health at the enrollment of MMT [23] due to their history of drug use. In our study, this included people living with HIV, cancer survivors, and people living with chronic pain. They reported significant improvements in health, including reduced withdrawal syndrome, alleviated pain, and enhanced sleep quality. These positive changes can be attributed to reduced drug use and less high-risk behavior after receiving MMT. Additionally, the health education provided by MMT clinics likely increased their awareness of health issues, resulting in more proactive health-seeking behavior [24]. Furthermore, as a vulnerable group for mental health issues, they experienced a decrease in negative emotions and an increase in self-confidence after being treated. Better health status facilitated their reintegration into society [25], leading to closer family relationships and more employment opportunities. According to Sun et al. [26], the overall employment rate of MMT participants increased from 26.4% at baseline to 41.6% after 6-month treatment and to 59.8% after 12 months. Employment not only alleviated their financial burden but also contributed to improved family relationships and a sense of fulfillment, as indicated by our findings.

Hence, it can be concluded that the effectiveness of MMT extends beyond the realm of individual health improvement. It encompasses various interconnected benefits, including enhancements in health status, interpersonal relationships, and social functioning. These mutually reinforcing advantages create a virtuous circle, leading to an overall improvement in quality of life.

In terms of clinical treatment, participants in this study reported frequent changes in their daily methadone dose during the long-term treatment. Participants with cancer, or pain, or those taking HIV antivirals tended to increase their dose. Methadone has pain-relieving effects and its metabolism can be accelerated by HIV antivirals, necessitating higher doses for these individuals [27]. However, almost every participant expressed a desire for dose tapering and took action toward full recovery from opioid dependency. This is consistent with previous studies, which indicated that around 41–57% of participants tapered their doses [28, 29]. According to our findings, the frequency, speed, and duration of dose tapering varied among participants, with those on relatively higher doses, such as over 100 mg per day, being more adaptable to larger decreases. People who added doses due to temporary illness would decrease the dose as usual once they felt better. Participants on lower doses, such as 5–10 mg per day, found it challenging to adjust to the side effects, particularly insomnia if they decreased the dose too much or too quickly. In light of these findings, it is important to establish official dose adjustment guidelines for Chinese participants, considering individual adaptation [30].

Apart from all the improvements, participants also highlighted the challenges they faced throughout their treatment, including conflicts between the opening hours of clinics and working schedules, the fear of treatment discontinuation, economic hardship, and the lack of dose adjustment guidelines and discrimination.

Another challenge was that most MMT clinics had opening hours that overlapped with normal working hours. This made it difficult for participants to arrive at work on time and limited their job options. This conflict exacerbated their economic hardship as they had to choose jobs near the clinic or opt for night shifts to maintain the treatment. Participants may be more likely to drop out of MMT due to this conflicted schedule [29].

Although negative effects such as decreased clinic visits and increased levels of anxiety and depression among participants were reported when COVID-19 first broke out in 2019 [31], a limited impact was reported since the pandemic was relatively well-controlled in Guangdong, especially in the less-populated areas. Most clinics opened as usual, and measures such as medication delivery by police officers or community workers were implemented for those in quarantine [32]. Other countries, however, tended to liberalize the taking-home policy as the response [33, 34]. Despite the limited impact, challenges relating to treatment discontinuity and its negative impact on income emerged as the most significant concerns, as revealed by our findings. These challenges also triggered participants’ pre-existing mental disorders [35]. Additionally, emergencies such as hospital admissions could disrupt the continuity of MMT. The current policies and processes for accessing medication vary across regions, and participants expressed complaints about the cumbersome process of applying for dose delivery to the hospital. Hence, there is an urgent need to develop official guidelines or manuals for the preparedness for emergencies that are feasible and accessible for both participants and staff.

Moreover, discrimination from employers was also reported and caused economic hardship. Participants shared their experiences of being refused employment due to their identification as PWUD; meanwhile, the unemployed participants were more likely to experience discrimination from the public [36]. This contradiction further hindered the progress of their return to normal life. All of the above indicates that economic hardship is a priority challenge to be addressed for this group [37, 38], as they sought a more comprehensive return to normal life, beyond simply being abstinent from addiction. Therefore, targeted interventions should primarily focus on helping long-term participants resume social functioning. Simultaneously, improving adherence to MMT should remain a key area of focus for short-term participants.

To address all the challenges identified, our participants provided several possible solutions. For MMT clinics, they suggested that the opening hours should be extended or staggered to accommodate commuting time, ensuring both timely treatment and arrival at work. Another expectation of participants was to lower the cost of treatment, or even provide it for free, considering their financial hardship.

In term of improving the effectiveness of MMT, they emphasized the importance of health education and thought it should be integrated to the daily treatment to help the peer develop a proper understanding of MMT, reducing relapses and dropouts. As for the discontinuation of treatment, they believe narcotics control departments or CDC should develop comprehensive official guidelines to respond to emergencies, or simplify the current process, making it more feasible and operable for both clinic staff and participants.

They also expect a platform for job hunting without disclosing their drug use history. Providing a basic living allowance or offering jobs to eligible people should also be considered to alleviate their financial burden, based on the participants.

In summary, the clinic could improve the flexibility of opening hours and continue related interventions to ensure the effectiveness of MMT; narcotics control departments or CDC are expected to developed a feasible and simple process for MMT under emergency; relevant public sectors are responsible to create more jobs or individualized supporting plans for participants experiencing poverty.

## Strengths and limitations

To our knowledge, this study was the first attempt to explore the improvements and challenges experienced by MMT participants, considering the changing drug situation in China during the past two decades. The perspectives of participants who have received an average of 12 years of treatment could be used as comprehensive and representative references for further policy-making. However, this study has several limitations. Firstly, the interviewed participants all came from Guangdong province, and the study would benefit from increased representation by recruiting individuals from other regions, especially those with limited resources, to explore additional challenges with MMT uptake. Secondly, service providers and other stakeholders were not included in the interviews, and their insights are also critical for the overall improvement of MMT. Nevertheless, the participants provided suggestions for both clinics and relevant government sectors, which can serve as a basis for comparison and reference for further studies. Short-term participants also need to be considered in future research as they may have different treatment experiences and their views might be still helpful in improving the MMT program.

## Conclusions

The improvement in health status, family relationships, and social functioning were the most positive changes experienced by participants who received long-term MMT. There is an urgent need to develop official guidelines for dose adjustment and treatment guarantees in emergencies to improve the quality and accessibility of the treatment. Strategies like health education, employment promotion, and clinic opening time adjustment should be also prioritized for a better return to society for long-term participants. It is also essential to establish a follow-up mechanism for participants no longer on MMT to reduce the likelihood of relapse to heroin and other illicit narcotics.

### Supplementary Information

**Additional file 1: Table S1.** Suggestions for MMT services from the perspective of participants. 2. Topic guide for MMT participants’ experience during the treatment. 3. Informed Consent for Participants of Methadone Maintenance Treatment Interview.

## Data Availability

The data analyzed in this study are not publicly available to protect the confidentiality and privacy of participants. Reasonable requests may be considered according to the corresponding author.

## Abbreviations

CDC: Center for diseases control and prevention
HCV: Hepatitis C virus
HIV: Human immunodeficiency virus
IPA: Interpretative phenomenological analysis
MMT: Methadone maintenance treatment
PWUD: People who use drugs
PWUH: People who use heroin
