# Supplementary material for: Benefits and challenges experienced by participants on long-term methadone maintenance treatment in China: a qualitative study
Source: BMC Med. 2024 Jan 8;22:18. doi: 10.1186/s12916-023-03203-z (PMC10773040; doi:10.1186/s12916-023-03203-z)
Supplement: Supplementary file 1 — Additional file 1: Table S1. Suggestions for MMT services from the perspective of participants. 2. Topic guide for MMT participants’ experience during the treatment. 3. Informed Consent for Participants of Methadone Maintenance Treatment Interview. [file 12916_2023_3203_MOESM1_ESM.pdf]

Additional file

1.Table S1. Suggestions for MMT services from the perspective of participants

2. Topic guide for MMT participants' experience during the treatment

3. Informed Consent for Participants of Methadone Maintenance Treatment Interview

Table S1. Suggestions for MMT services from the perspective of participants

Institution	Suggestions
MMT clinics	• Adjust or prolong the opening times of the MMT clinics<br>• Reduce the cost or provide the treatment for free<br>• Enhance the public reputation of MMT
Narcotics control departments/CDC	• Develop official guidelines to ensure treatment continuation in exceptional circumstances
Other public sectors	• Apply for a basic living allowance for participants in need<br>• Create platforms or mechanisms to provide jobs for participants without disclosing their drug use history

Topic guide for MMT participants' experience during the treatment

Clinic:

Treatment number:

Age:

Duration of treatment:

Hello! We are a research team a of Sun Yat-sen University. It's great to have you with me today. I would like to ask you some questions about your participation experience during methadone maintenance treatment, which may last about 30 minutes. (Start to interview only when consent given.)

- **Experience of MMT**

1. When did you attend MMT? Who and what reasons brought you here?

2. What were main changes you feel about after being treated?
   (**Prompts**: improved the health, family relationships or back to a normal life, etc.)

3. Have you ever relapsed during treatment? If so, what was the cause and why did you choose to return to MMT?
   (**Prompts**: Inadequate dose? Financial burden? Moving to other places? Illness?)

4. How much methadone do you currently take everyday? Have you ever asked a dose adjustment and why?

5. How long did dose adjustment phase last and how did you feel about it?

6. Are you still on the dose adjustment? If not, what made you end this process?

7. Would you be willing to taper dose if you could and can you tell me why?

- **Changes in the number of MMT participants**

1. Do you notice the change in **number of MMT participants** of this clinic during recent years? What did it change and what lead to the changes
   (**Prompts**: Are people you know still on MMT? If not, why?)

2. (If answered "less and less") At which stage, did they choose to quit MMT and what is their current status?
   (**Prompts**: Death, Relapse, and Referral)

3. Do you have any intention to quit as well? What would be the reason and how long do you need to achieve this goal?

- **Changes brought by COVID-19 epidemic**

We just talked about your treatment experience, let's now think about the impact of COVID-19 epidemic.

1. Did COVID-19 epidemic affected your treatment and how did it affect?

(**Prompts**: Closure of clinics/ Inadequate supply of medication/ Travel restriction resulting in interruption of treatment? / Less contact with drug users?)

(If answered 'no impact', skip to question 3)

2. What was the clinic's response if your treatment was affected? Did that solve the problem?

3. Did your dose change because of the epidemic? How did it change and why do you want it to be changed?

4. Did your treatment return to normal now, or has it remained? Could you tell me the details about it?

5. Can you tell me that how you felt about during the epidemic or the lockdown, and what did you mostly worried about?

(Prompts: Was the negative emotion triggered, especially when there was lockdown?)

6. Before the epidemic, did you previously experience anxiety, depression and other emotions before? how long did you have it and what caused it?

7. Did such issues affect your treatment?

(**Prompts**: dose increase/ withdrawal symptoms/ taking medication for mental issues)

8. Do your friends or people you know (other participants) have such issues? What are the usual causes?

9. If so, did you or your friends feel these issues being trigger during COVID-19 epidemic? What were specific reasons and symptoms if did?

10. What other services are provided in your clinic? Are they still being provided after the epidemic? What is your view on these services?

    (**Prompts**: peer education/HIV testing/psychological counselling, etc.)

11. Were these services affected during the epidemic and what are the specific impacts?

Challenges experienced in the process and suggestions

1. What are the biggest challenges you personally encountered in the process of MMT? Could you give me 1 or 2 examples?

(**Prompts**: high costs, long travel distances, cumbersome reimbursement procedure)

2. What can be done to solve these problems in your opinion?

3. What do you think are the biggest difficulties or problems that this community are facing in their daily lives?

(**Prompts**: discrimination/financial burden/other)

- **Possible solutions**

We talked about some challenges and problems, let's talk about their possible solutions.

4. Do you think there are possible solutions to these problems, both about treatment or the personal life for this community?

5. Do you have any other suggestions for MMT clinics to improve the treatment quality?

6. What else should be done by community, narcotics control offices or other sectors to help with you in general?

Informed Consent for Participants of Methadone Maintenance Treatment Interview

Hello, dear friend!

We are a research team from school of public health at Sun Yat-sen University. We would like to know your experience about methadone maintenance treatment. The interview may to take about 30 minutes. Please read the following information carefully and sign at the end of the document if you agree to attend. We greatly appreciate your insightful opinions which could contribute to the improvement of MMT.

1. This interview will be audio-recorded and transcribed into text.
2. The information you provide will only be used by members of our research team for academic purpose and will never be shared with others for any other purposes.
3. This interview will be anonymous and we promise that the confidentiality of your data is strictly ensured which means it would not be disclosed or used for other purposes.
4. The data relating to this interview will be backed up and stored by Sun Yat-sen University.
5. You can choose pause or withdraw from the interview at any time during the process if you feel uncomfortable.
6. If you decide to withdraw after the interview, please inform us in 3-5 days, otherwise you cannot withdraw since your data would have been analyzed already.

I am aware of the above content and agree to participate in the interview.

Participant’s Signature: ______________ **Date: ___________________**

Researcher’s Signature: ______________ **Date: ___________________**
